# A rare case of a pedunculated skin tag

**DOI:** 10.11604/pamj.2025.52.152.46461

**Published:** 2025-12-09

**Authors:** Mayur Pradip Mhaske, Amol Deshpande

**Affiliations:** 1 Department of Rachana Sharir, Mahatma Gandhi Ayurved College Hospital and Research Centre, Datta Meghe Institute of Higher Education and Research (Deemed to be University), Salod (H), Wardha, India

**Keywords:** Skin tag, pedunculated mass, popliteal fossa

## Image in medicine

A 41-year-old male patient presented with a two-month history of a progressively enlarging, pedunculated mass in the medial border of lower side of popliteal fossa of the left leg. The lesion was fleshy, skin-colored, tender, movable, and had mild discomfort when palpated. It was 4 cm in diameter. The patient mentioned no systemic symptoms, and no more abnormal findings were seen during the clinical assessment. The differential diagnosis includes acrochordon, skin tag, and soft fibroma. Skin tags, also known as acrochordons, are common benign growths in regions where skin rubs against skin or clothing, such as the neck, armpits, and groin. They are normally soft and harmless, ranging from a few millimeters to several centimeters. Skin tags are made up of loose collagen fibers and blood vessels that are surrounded by the epidermis and are frequently pedunculated. While they are usually asymptomatic, they can be irritated if they get trapped in jewelry or clothing.

**Figure 1:**
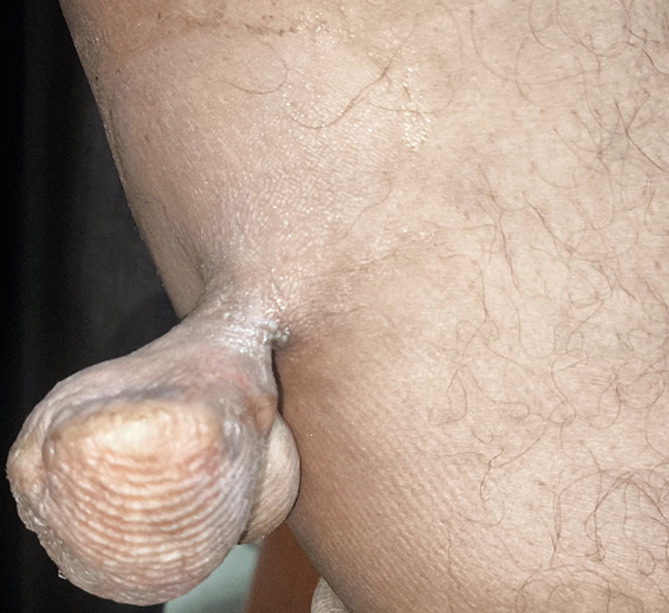
pedunculated skin tag

